# *GEMMI* and *Servalcat* restrain *REFMAC*5

**DOI:** 10.1107/S2059798323002413

**Published:** 2023-05-05

**Authors:** Keitaro Yamashita, Marcin Wojdyr, Fei Long, Robert A. Nicholls, Garib N. Murshudov

**Affiliations:** a MRC Laboratory of Molecular Biology, Cambridge Biomedical Campus, Francis Crick Avenue, Trumpington, Cambridge CB2 0QH, United Kingdom; b Global Phasing Ltd, Cambridge, United Kingdom; Diamond Light Source, United Kingdom

**Keywords:** *REFMAC*5, *GEMMI*, *Servalcat*, model refinement

## Abstract

Macromolecular refinement uses experimental data together with prior chemical knowledge (usually digested into geometrical restraints) to optimally fit an atomic structural model into experimental data, while ensuring that the model is chemically plausible. In the *CCP*4 suite this chemical knowledge is stored in a Monomer Library, which comprises a set of restraint dictionaries. To use restraints in refinement, the model is analysed and template restraints from the dictionary are used to infer (i) restraints between concrete atoms and (ii) the positions of riding hydrogen atoms. Recently, this mundane process has been overhauled. This was also an opportunity to enhance the Monomer Library with new features, resulting in a small improvement in *REFMAC*5 refinement. Importantly, the overhaul of this part of *CCP*4 has increased flexibility and eased experimentation, opening up new possibilities.

## Abstract 2

The restraint-generation part of the macromolecular atomic model-refinement program *REFMAC*5 has been delegated to *GEMMI*. A controller program was implemented in *Servalcat* to distribute tasks between *GEMMI* and *REFMAC*5.

## 1. Introduction

The *CCP*4 software suite (Winn *et al.*, 2011 ▸; Agirre *et al.*, 2023 ▸) is an ecosystem of software projects: programs, libraries, data stores and graphical user interfaces. Many of the programs and libraries have been developed by only one scientist. In such cases it is beneficial to limit the scope of the project in order to ease maintenance. Additionally, programs with narrower scope are easier to substitute. The result is that *CCP*4 often has more than one program available to perform the same (or a similar) task. Newer programs with the same functionality often co-exist with older ones and, in time, make them obsolete. This creates a healthy ecosystem.

In the 1980s, the main atomic structure refinement tool in *CCP*4 was the least-squares refinement program *PROLSQ* (Hendrickson, 1985 ▸). The stereochemical restraints used by *PROLSQ* were generated by a separate program called *PROTIN*, which had its own internal library containing information about amino acids, common ligands and cofactors. They were mostly used for the refinement of atomic structure models of proteins. In the 1990s *PROLSQ* was obsoleted by the maximum-likelihood refinement program *REFMAC* (Murshudov *et al.*, 1997 ▸), which initially also relied on *PROTIN*. In the early 2000s, *PROTIN* was replaced by a more general and extendable Monomer Library (CCP4-ML; Vagin *et al.*, 2004 ▸) and a software tool *MAKECIF*; the latter became an internal part of *REFMAC*5. *MAKECIF* parses the CCP4-ML, analyses the model, prepares restraints involving atomic positional parameters with respect to their neighbours and calculates the positions of riding hydrogen atoms using simple geometric rules.

Other popular macromolecular refinement programs, such as *phenix.refine* (Afonine *et al.*, 2012 ▸) and *BUSTER* (Bricogne *et al.*, 2017 ▸), are distributed with their own restraint libraries. The organization of these libraries differs from that of the CCP4-ML to a greater or lesser extent. Here, we will only discuss resources and implementations within the *CCP*4 suite.

Over the years *REFMAC*5 has grown in features, becoming one of the largest and most complex programs in *CCP*4. Recently, we started an effort to slim it down by delegating some of the functionality to two other software tools: *GEMMI* (Wojdyr, 2022 ▸) and *Servalcat* (Yamashita *et al.*, 2021 ▸). The intention is to limit the scope of *REFMAC*5 to actual model refinement and prepare for further evolution of the *CCP*4 suite.

*GEMMI* is a toolkit for crystallographic computing developed as a joint effort between Global Phasing Ltd and CCP4. It contains a variety of functions for working with structural models, diffraction data and density maps.

*Servalcat* is a pipeline that was originally designed for single-particle cryo-EM structure refinement, leveraging *REFMAC*5 as the underlying refinement engine, and for the calculation of sharpened and *F*_o_ − *F*_c_-type difference maps. *Servalcat* has now been extended for application in macromolecular crystallography.

In this contribution, we report details of new developments in *GEMMI* and *Servalcat* that manage the preparation of restraints for macromolecular refinement. These developments replace a sizable portion of *REFMAC*5, removing some of the limitations, providing a couple of improvements and faster operation (although the effect on the total refinement time is not significant). It is also easier to extend, which means that new ideas for restraining atomic structure models can easily be implemented and tested. In addition, we report an enhancement to the CCP4-ML that was introduced during this overhaul: link-related aliases.

## 2. CCP4 Monomer Library and link-related aliases

The organization of the CCP4-ML has been described by Vagin *et al.* (2004 ▸) and further summarized by Lebedev *et al.* (2012 ▸), and the recent expansion has been detailed by Nicholls *et al.* (2021 ▸). Vagin and coworkers called it the ‘REFMAC5 dictionary’, Lebedev and coworkers called it the ‘CCP4 template-restraints library’ and Nicholls and coworkers called it the ‘CCP4 Monomer Library’; these all refer to the same data store of dictionaries and associated metadata that is distributed as part of the *CCP*4 suite. Currently, the CCP4-ML dictionaries are based on a statistical analysis performed by *AceDRG* (Long *et al.*, 2017 ▸) using small-molecule structure models from the Crystallography Open Database (Gražulis *et al.*, 2012 ▸).

The CCP4-ML includes almost all ligands and macromolecular constituent blocks available in the Chemical Component Dictionary (CCD; Westbrook *et al.*, 2015 ▸) from the Protein Data Bank (PDB; wwPDB Consortium, 2018 ▸). Monomer descriptions in the CCP4-ML have the same atom names and bond types as in the CCD, but they additionally contain ideal values and standard uncertainties associated with bond lengths, angles, torsion angles, planarities and chiralities: this information can be used directly for the generation of restraints for use in atomic structure refinement.

Alongside monomer descriptions, the CCP4-ML also contains *links* and *modifications*. When two monomers form a covalent bond, the corresponding link specifies which additional restraints should be applied and which modifications should be prescribed to the bonded monomers as a consequence of covalent bond formation. A modification (in the context of the CCP4-ML) is a predefined set of changes that are applicable to a monomer description. For example, the monomer description of alanine has an ideal C—O bond distance equal to 1.25 Å. When alanine forms a peptide bond with the next amino-acid residue in the polypeptide chain, the alanine is prescribed a modification (named ‘DEL-OXT’) that, among other changes to the description of this monomer, sets the ideal C—O bond length to 1.23 Å.

Links are either defined for bonds between specific monomers (for example for a disulfide bond between two cysteines) or, more generally, for bonds between monomers that belong to specified *groups*. Each monomer in the library belongs to one of the following groups: peptide, P-peptide (proline-like), M-peptide (*N*-methylated peptide), DNA, RNA, pyranose, ketopyranose, furanose or non-polymer (*i.e.* everything else). This allows, for example, the definition of a link between any ketopyranose and any pyranose, or a link between any peptide and an NH2 monomer.

**Figure 1:**
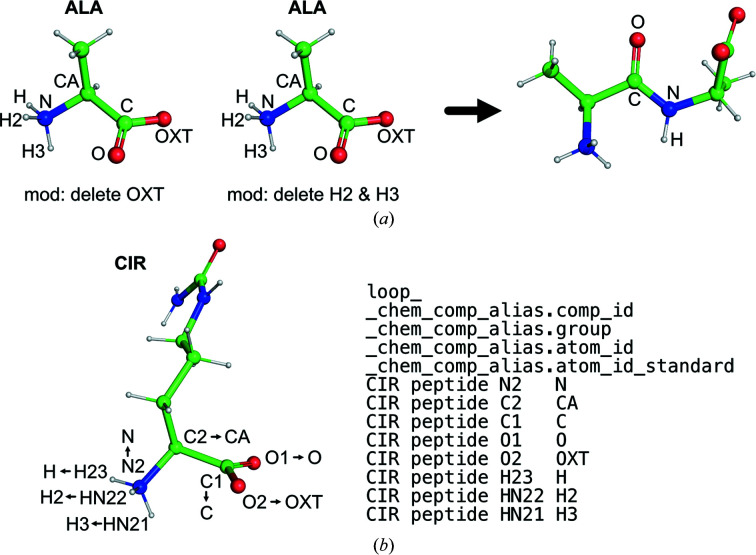
Link mechanism and alias. (*a*) In peptide linking (TRANS/CIS), OXT is deleted from one side and H2 and H3 are deleted from the other side, and a bond between C and N is then defined together with other restraints around this bond. The standard atom names are shown. (*b*) The peptide monomer CIR has equivalent main-chain atoms with different names. The alias table (_chem_comp_alias) specifies how their names should be changed in order to correctly use TRANS/CIS links. Images were created using *PyMOL* (version 2.4; Schrödinger).

Links and modifications rely on atom names. For example, TRANS and CIS links are defined for peptide monomers, which create a bond between C and N atoms with the removal of OXT and H2/H3 atoms from the C- and N-terminal sides, respectively (Fig. 1 ▸ *a*). These links and associated modifications set and modify several restraints between atoms named N, CA, C, O and H. Thus, the current link mechanism only works correctly if the atom names follow the standard nomenclature. However, as of November 2022, out of over a thousand types of monomer that form part of a polypeptide chain in the PDB, hundreds do not follow the standard atom-naming convention. A similar situation is found for monomers in polynucleotide chains. For this reason, many nonstandard amino-acid and nucleic acid monomers have had to be demoted to the non-polymer group.

To allow monomers with nonstandard atom names to be linked using the standard link descriptions we have introduced a mechanism of aliases. Monomer descriptions may now have a new category: _chem_comp_alias. This allows the definition of a correspondence between atom names in the monomer and those in the specific group, allowing a translation of atom-naming conventions. For example, an alias table has been added to the chemical component CIR (citrulline), which is a peptide monomer with a noncanonical main-chain atom nomenclature (Fig. 1 ▸ *b*). The alias table allows *GEMMI* to apply the standard polymer links and generate the corresponding restraints (see §3.1).

The implemented mechanism provides a workaround for nonstandard atom nomenclature, but the naming for the same monomer, in particular the naming of the leaving atom, still needs to be consistent. An inconsistency may occur when link-forming atoms in an isolated monomer are chemically equivalent. For example, nucleotide monomers have three oxygen atoms on the phosphate group, with the standard nomenclature OP1/OP2/OP3. Upon polynucleotide formation one of the oxygen atoms leaves and is replaced by the O3′ atom of the previous nucleotide. For a number of monomers (examples include AAB and F86) the choice of the leaving atom is not consistent across the PDB. Static aliases cannot help with such inconsistencies.

### 2.1. Example of alias usage in refinement

**Figure 2:**
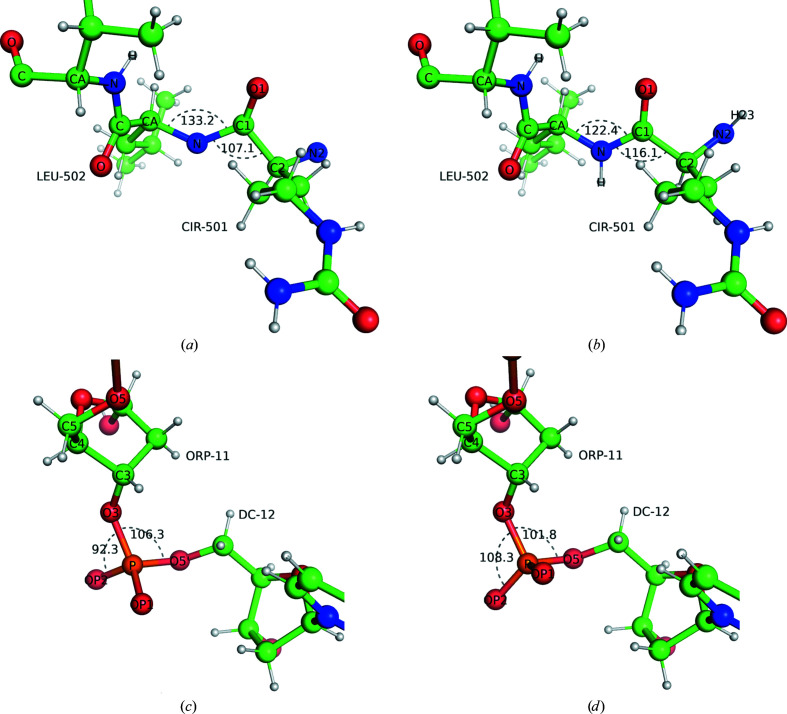
(*a*, *b*) PDB entry 1ol1 and (*c*, *d*) PDB entry 3waz after 20 cycles of geometry optimization using the *Servalcat REFMAC*5 controller (*a*, *c*) without and (*b*, *d*) with the alias mechanism. Since the monomers CIR (*a*, *b*) and ORP (*c*, *d*) do not follow the standard nomenclature, their chemistry is not properly recognized without the alias mechanism (missing hydrogen atoms in peptide bonds and missing restraints other than bond length). The ideal bond angles in the CCP4-ML are CA—C—N, 115.9°; C—N—CA, 122.1° (for a peptide link); O3′—P—OP2, 108.5°; O3′—P—O5′, 101.1° (for a phosphodiester link). Images were created using *PyMOL* (version 2.4; Schrödinger).

We demonstrate the usage of an alias table using CIR (citrulline) contained in the PDB (PDB entry 1ol1) as an example (Figs. 2 ▸ *a* and 2 ▸ *b*). Using the CCP4-ML with and without alias tables, geometry optimization was performed using the *Servalcat REFMAC*5 controller (see §3.3). With the alias, the TRANS link restraints were applied automatically to the peptide bonds on both sides of the CIR residue. Single hydrogen atoms were added and the whole geometry was maintained properly. Without the alias, if the user does not make extra effort to generate link descriptions for CIR–peptide and peptide–CIR bonds using an external program, only the bond-length restraints are applied automatically, providing that the model contains a corresponding LINK record (see §3.1). In the latter case, the geometry around linked atoms can easily be distorted; it is important to ensure that comprehensive dictionaries are always used rather than the simple fallback solution.

We performed the same test for a DNA case using ORP (2′-deoxy-5′-phosphonoribose) from PDB entry 3waz (Figs. 2 ▸ *c* and 2 ▸ *d*). Similarly, the full link description, applied through the atom name aliases, restrained the geometry around the phosphodiester bonds and avoided the distortion of angles.

## 3. *GEMMI*fication of *REFMAC*5

*REFMAC*5 has an internal mechanism called *MAKECIF*, which maps stereochemical information from the CCP4-ML to the current atomic model for the purpose of generating restraints. It can also generate riding H-atom positions using local geometry. The restraints and H-atom positions are then passed to *REFMAC*5 for restrained refinement. Additional restraints, including nonbonded interactions, are calculated on the fly at every cycle of refinement, utilizing information about the chemical types of the atoms involved in interactions.

Although this mechanism has worked for 20 years, producing reasonable results, adding new features has become increasingly difficult and it is limited in how it handles alternative conformations of monomers. In particular, it cannot process microheterogeneities (point mutations) and partial bonding (for example when only one of two conformers forms a bond to the next residue). This functionality has now been implemented in *GEMMI*, overcoming these limitations, and *REFMAC*5 has been modified to take this information from an externally generated file. In addition, a controller program has been implemented to hide this change from the user, as described in §[Sec sec3.3]3.3. The new refinement setup can be run from the command line by replacing the traditional *REFMAC*5 invocation with one word: refmacat XYZIN …

### 3.1. Link identification

One of the steps in restraint preparation is finding link descriptions for covalent bonds in the structure. Here, we outline this process.

Firstly, we need to determine covalent bonds between residues. Bonds between consecutive residues in a polymer are implicit. Other bonds should be explicitly listed in appropriate records in the input coordinate file (LINK/LINKR and SSBOND records in PDB format, _struct_conn in mmCIF format).

Alternatively, bonds can be determined automatically from the interatomic distances in the model. This option is turned off by default and for the purposes of this section we assume that it is not used.

The LINKR record is a nonstandard extension of the PDB format used in *CCP*4. In mmCIF format, the corresponding extension is _struct_conn.ccp4_link_id. These extensions allow the user to provide the name of the link description. If the name is provided, no further identification is necessary.

The presence of a bond between sequential residues in the polymer is determined from the distance between the atoms that can form the bond. The cutoff distance and the atom names used in this determination depend on the polymer type. Additionally, the atom names can be changed by the alias mechanism (note the C1—N bond in Fig. 2 ▸ *b*). The distance-based check can be overwritten by explicit LINK/LINKR records. In particular, a LINK record for two sequential residues means that a bond is present, and a LINKR record with the name ‘gap’ means that the bond is absent.

If the link name is not provided, the bonds are matched to all link descriptions from both the CCP4-ML and user-provided dictionaries, and the best-matching, most specific link is used. This procedure is applied to both polymer bonds and bonds from the LINK records.

The link specificity depends on how the linked residues are defined. A hypothetical LYS–PRO link is more specific than peptide–PRO, which in turn is more specific than peptide–P-peptide, while peptide–peptide is the least specific. The monomer group can be matched using an alias table if the monomer description contains such a table. For some bonds, more than one description is provided with the same specificity (examples include TRANS and CIS links for peptide bonds and ALPHA1-4 and BETA1-4 links for glycosidic bonds). In such cases, the chirality and the ideal values of torsion angles from the link description are compared with the values in the atomic model and the best-matching link is used.

If no matching link description is found, only the bond length is restrained, based on the chemical types of the bonded atoms.

The whole procedure accounts for the possibility of alternative conformations, which makes it much more complex. It is possible for a C atom in an amino acid to form peptide bonds to two alternative locations of the N atom in the next residue. The link description required for both bonds can be different (for example, CIS and TRANS). Alternatively, the next residue can be microheterogeneous and the nitrogen atoms in two conformations can have different names (example: KOR/K1R microheterogeneity in PDB entry 2ci1 with names N and N2). As another alternative, the next residue can be microheterogeneous but nevertheless there can be only one N atom that is shared by both conformations (as an example, in PDB entry 1ejg PRO/SER microheterogeneity is modelled with a shared N atom; this atom is formally in PRO, but is linked to alternative CA atoms from both PRO and SER). Other interesting cases include partial cleavage (one conformation forms a link and the other is a terminus; for example PDB entry 6ozo) and a LINK record specifying atoms with different altloc indentifiers (for example PDB entry 2e7z).

### 3.2. Positions of riding hydrogen atoms

Similarly to *MAKECIF*, *GEMMI* can add hydrogen atoms to a model, which are placed in riding positions. The position of a riding hydrogen atom is calculated from the CCP4-ML restraints. A hydrogen atom must be away from its parent at a distance equal to the ‘ideal’ distance and it must obey chirality and planarity rules. Angles involving generated hydrogen atoms should also be as close as possible to the ‘ideal’ values.

If the riding position cannot be determined unambiguously, *i.e.* if there are multiple positions that obey all conditions (for example an H atom on a hydroxyl group) or the protonation state is unknown (for example H^δ1^ and H^ɛ2^ of histidine), the hydrogen atom is placed at one of the possible positions and its occupancy is set to zero (in the current implementation). *REFMAC*5 ignores such zero-occupancy atoms during refinement.

The electron cloud centre of the hydrogen atom can be noticeably shifted from the nucleus position. Which of the two we want to use depends on the experimental technique. X-rays interact with electrons. Neutrons interact with nuclei. Electrons interact with the electrostatic potential, which is formed by electron density and nuclei charges: when refining against electron scattering data the positions of both the nuclei and the electron clouds are needed (Yamashita *et al.*, 2021 ▸). This means that we need to know two distances between a hydrogen atom and its parent atom: the distance between the electron cloud centres and the distance between the nuclei. Originally, the CCP4-ML contained only the former. The latter was added recently (Nicholls *et al.*, 2021 ▸) and is now supported in *REFMAC*5 refinement (Catapano *et al.*, in preparation).

### 3.3. *Servalcat* as a *REFMAC*5 controller

**Figure 3:**
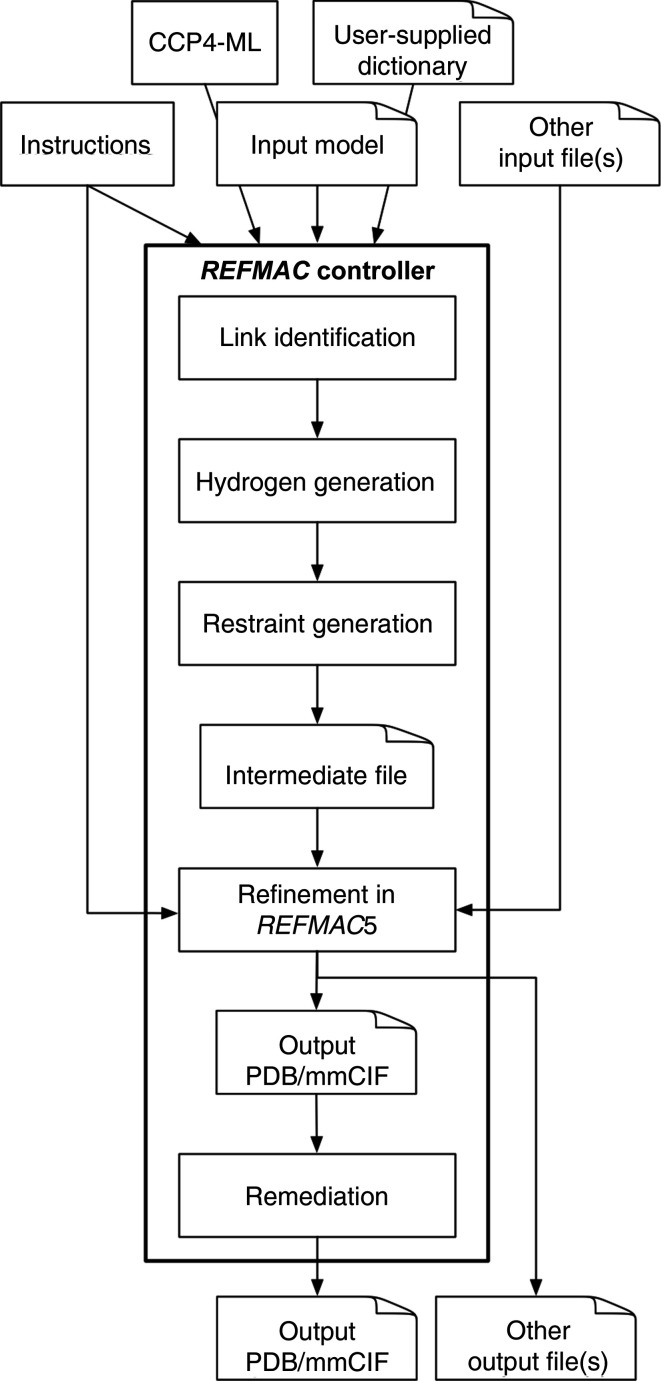
The workflow of the *REFMAC*5 controller implemented in *Servalcat*. It reads instructions related to *MAKECIF*, generates hydrogen atoms (if requested) and generates restraints. After *REFMAC*5, PDB/mmCIF files are rewritten.

*Servalcat* has been extended to control the flow of information between *GEMMI* and *REFMAC*5 (Fig. 3 ▸). It is designed to have the same interface as *REFMAC*5, and is available as a standalone executable (command refmacat). *Servalcat* takes *REFMAC*5 input keywords, runs *GEMMI* with appropriate sets of instructions and then provides the *GEMMI*-generated intermediate files (which contain modified coordinates and restraints) to *REFMAC*5, along with instructions relating to refinement and map calculation. After *REFMAC*5 finishes a refinement session, the output coordinate files are amended. If the structure cannot be written in the standard PDB format, popular extensions are employed: two-letter chain IDs and the hybrid-36 encoding (https://cci.lbl.gov/hybrid_36/) for atom serial numbers and residue sequence numbers.

## 4. Conclusions and outlook

New functionalities have been implemented in *GEMMI*: the ability to read input coordinate files, perceive the internal chemical structure of macromolecules (polymers, monomers, links and necessary modifications) and map appropriate restraints from the CCP4-ML to an atomic structure model. The resulting restraints are accepted by *REFMAC*5 for use in restrained refinement. A smooth transition is facilitated by *Servalcat*, which controls the overall workflow and communication between *GEMMI* and *REFMAC*5, and provides the user interface.

While replicating the results produced by *MAKECIF* in *REFMAC*5, the implementation in *GEMMI* has been designed to increase flexibility, extensibility and maintainability. However, there is still room for improvement in restraint generation. One of the planned improvements is the introduction of coordination- and chemistry-dependent metal link restraints.

Nonstandard atom nomenclature has been causing problems with linking peptide/RNA/DNA monomers and associated restraint generation. This issue has been addressed by the introduction of the alias-table mechanism, which maps non­standard atom names to the conventional atom names as defined within the CCP4-ML. However, this mechanism still relies on the atom names and unnecessarily distinguishes between chemically equivalent atoms (for example OP1/OP2/OP3 atoms of nucleotides). In future, a general solution for dealing with covalent linkages between monomers could use chemical atom types, similar to that internally used within *AceDRG* (Long *et al.*, 2017 ▸), in order to allow the definition of linkages between functional groups.

## 5. Software availability

All software described in this paper is planned for distribution by *CCP*4. The components are also available from GitHub: CCP4-ML (LGPL licence) at https://github.com/MonomerLibrary/monomers, *GEMMI* (MPL licence) at https://github.com/project-gemmi/gemmi and *Servalcat* (MPL licence) at https://github.com/keitaroyam/servalcat. The features described here have been implemented in *REFMAC*5.8.0405, *GEMMI* 0.6.0 and *Servalcat* 0.4.0.
